# A New Approach to the Diagnosis and Treatment of Cardiovascular Diseases

**DOI:** 10.3390/pharmaceutics17091141

**Published:** 2025-08-30

**Authors:** Dorota Bartusik-Aebisher, Aleksandra Kotlińska, Katarzyna Koszarska, David Aebisher

**Affiliations:** 1 Department of Biochemistry and General Chemistry, Faculty of Medicine, Collegium Medicum, University of Rzeszów, 35-959 Rzeszów, Poland; dbartusikaebisher@ur.edu.pl; 2 English Division Science Club, Faculty of Medicine, Collegium Medicum, University of Rzeszów, 35-959 Rzeszów, Poland; ak117604@stud.ur.edu.pl (A.K.); kk117603@stud.ur.edu.pl (K.K.); 3 Department of Photomedicine and Physical Chemistry, Faculty of Medicine, Collegium Medicum, University of Rzeszów, 35-959 Rzeszów, Poland

**Keywords:** cardiovascular diseases, nanotechnology, nanocarriers, nanoparticles in CAD diagnostics

## Abstract

Cardiovascular diseases (CADs) have long been considered the domain of the elderly. However, the prevalence of modifiable risk factors has led to their increased diagnosis in younger people. Conventional treatment methods offer a wide range of drugs with different mechanisms of action, but their use brings only limited benefits. Often occurring, persistent adverse effects encourage the abandonment of regular drug administration and thus prevent effective therapy. Methods are sought that allow for targeted, more precise drug delivery that would eliminate their systemic and toxic effects. Therefore, one of the areas of particular interest in the field of cardiovascular diseases is the topic of drug delivery systems based on nanotechnology. We reviewed articles in the PubMed database focused on the latest reports of the use of nanotechnology in the treatment of CADs. Results: Nanoparticles (NCs) bring about many benefits compared to conventional preparations, which results from the possibility of carrying a larger amount of functional cargo and directing the therapy to individual cellular targets, as well as increasing the bioavailability of the transported drug. The introduction of NC to CAD treatment may allow for more effective therapy, but most importantly, it provides an opportunity for faster and more accurate diagnosis of developing disorders at a stage when they do not yet produce symptoms. Nanotechnology, thanks to the enormous variety of designed structures and functions, shows exceptional potential in the diagnosis and treatment of cardiovascular diseases. However, its widespread implementation in clinical practice is a significant challenge. Further research is necessary to provide reliable data on the pharmacokinetics, toxicity, and long-term safety of nanocarriers. The development of industrial methods for the production of nanocarriers with controlled and repeatable physicochemical properties while maintaining economic profitability remains a key challenge. Fulfilling these conditions is necessary for introducing nanotechnology as a standard method in modern cardiovascular medicine.

## 1. Introduction

Cardiovascular diseases (CADs) are a significant problem that burden the healthcare system. According to the latest data (Eurostat), in 2021, 1.71 million deaths were recorded in Europe due to CADs, which constitutes 32.4% of all deaths and significantly exceeds the percentage of the second most common cause of death—cancer (malignant tumors: 21.6% of all deaths) [1]. Among circulatory system diseases, the highest morbidity and mortality is related to coronary artery disease [2]. Despite the progress made in modern medicine, a significant increase in the number of morbidity and deaths from cardiovascular causes is visible. Every year, more than 20 million people die from CADs worldwide, and it is estimated that by 2030, this number will increase to 23.6 million [3]. This is mainly due to the increasing prevalence of modifiable risk factors, which include unhealthy eating habits, lack of physical activity leading to obesity and hyperlipidemia, and alcohol abuse and smoking. Chronic exposure to unfavorable risk factors with accompanying genetic predispositions results in the gradual development of hemodynamic disorders—most often against the background of increasing atherosclerotic changes and hypertension—and ultimately leads to the manifestation of symptoms of cardiovascular disease [4]. Conventional therapeutic treatment for circulatory system diseases includes the use of antithrombotic, antiplatelet, thrombolytic, and lipid-lowering drugs as well as invasive procedures, such as coronary artery bypass grafting or stent implantation. Despite the wide availability of drugs with different mechanisms of action, the implemented therapeutic treatment brings only limited benefits and only in some people suffering from CAD, and it causes side effects. Common disadvantages of available drugs include poor absorption, short plasma half-life, and high plasma clearance, which is why higher doses are necessary to achieve therapeutic concentrations, which are in turn associated with the intensification of systemic and toxic side effects [5].

**Figure 1:**
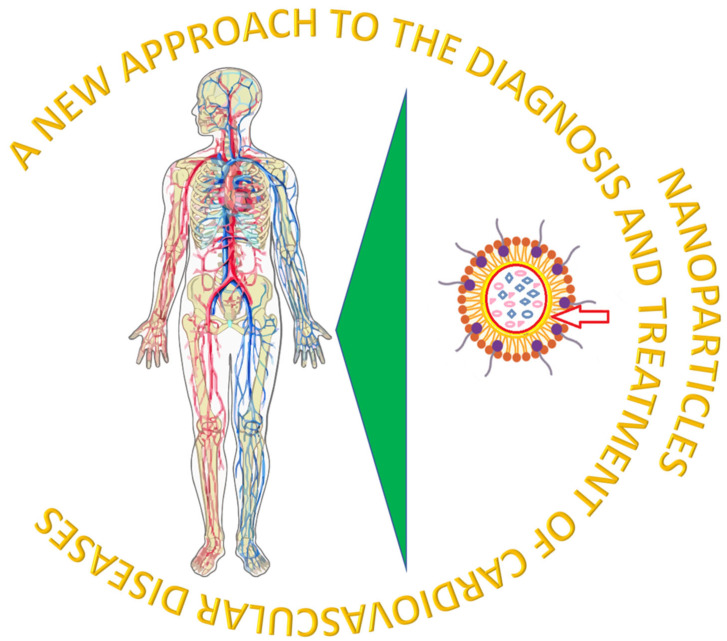
The profile of the use of nanoparticles for the diagnosis and treatment of cardiovascular disease.

Currently, one of the areas of particular interest in the field of cardiovascular diseases is the topic of nanotechnology-based drug delivery systems. This innovative field of science deals with the development of particles with dimensions of 1–100 nm, composed of biomaterials (e.g., lipids, proteins), metals, or combinations of these materials. Additionally, the surface of nanoparticles (NC) can be modified with markers (peptides, polymers, antibodies) that enable the delivery of transported substances to specific tissue targets or even to a specific subcellular compartment. In this way, the accumulation and retention of the drug in the diseased site are increased, while the systemic toxicity is limited [6]. Nanoparticles bring about many advantages compared to conventional preparations available on the market, which results from the possibility of the easier transfer of a larger amount of functional cargo and directing the therapy to individual cellular targets, as well as increasing the bioavailability of the transported drug. The introduction of NCs to CAD treatment may allow for more effective therapy, but most importantly, it gives the opportunity for the faster and more accurate diagnosis of developing disorders at a stage when they do not yet produce symptoms (Figure 1). Nanomaterials allow for the improvement in biosensors and imaging strategies at the molecular level, using techniques such as magnetic resonance imaging, optical imaging, or nuclear scintigraphy. Specially designed nanoparticles, such as liposomes, polymers (PLGA), inorganic nanoparticles (e.g., AuNP), natural NCs (HDL), or biomimetic nanoparticles (with a cell membrane coating), due to their unique physicochemical properties, act as targeted carriers of markers or drugs directing the active substance to pathologically changed sites. Additionally, some nanomaterials, due to their internal healing properties, such as antioxidant, anti-inflammatory, photoelectric, or photothermal effects, can be used independently as therapeutic agents. Increasing emphasis is being placed on developing technologies that allow for the introduction of the concept of theragnosis, meaning the combination of diagnostics with therapeutic action, into clinical practice. Thanks to this approach, it is possible to introduce more effective treatments more quickly, with reduced side effects and increased patient safety [7]. Creating effective and safe nanoparticles for use is a great challenge; making even small modifications to their shape, size, and surface structure can change the pharmacokinetics of these particles, meaning that precise production methods need to be used, thus driving up costs. Therefore, the aim of searching for new nanocarriers should not only be to make the treatment more effective but also to develop methods that allow for the use of nanoparticles on a large scale.

## 2. Pathophysiology of Atherosclerosis

Atherosclerosis is a chronic inflammatory disease of the arteries, commonly considered to be a disease primarily affecting the elderly. However, it is being diagnosed in younger people more frequently, which results from the increasing prevalence of risk factors such as smoking, hypertension, diabetes, or a diet rich in highly processed foods, as well as from the progress made in the development of diagnostic methods for detecting early atherosclerotic changes [8]. The formation of atherosclerotic changes is the result of a series of pathological processes that are initiated by the dysfunction of the endothelium of the arterial wall. The accumulation of low-density lipoproteins (LDLs) in the inner membrane and their subsequent oxidation lead to the activation of endothelial cells lining the arterial walls and the initiation of an inflammatory cascade. The secreted cytokines and chemokines facilitate the recruitment of monocytes, which migrate to the site of inflammation, where they differentiate into macrophages. These, in turn, phagocytize oxidized LDLs to form foam cells that secrete proinflammatory cytokines and growth factors, leading to further lipid accumulation and stimulating smooth muscle cell migration into the intima, which extends the pathological process. In the later stages of atherosclerosis, extracellular matrix components, including collagen, are deposited, resulting in the formation of a fibrous cap that stabilizes the atherosclerotic plaque [9]. However, as the plaque grows in size and necrotic lesions develop, the risk of plaque rupture increases, which can lead to life-threatening complications. The progression of atherosclerotic disease is not only associated with cardiovascular events such as coronary artery disease and ischemic stroke but also serves as an important prognostic factor determining the length and quality of life. The association between atherosclerosis and these conditions emphasizes the need for early diagnosis and implementation of effective therapeutic strategies [10]. Advanced high-resolution diagnostic methods using new biomarkers could enable the detection of atherosclerotic changes in asymptomatic individuals, which opens up the path of implementing effective and targeted treatments of fundamental importance for maintaining the health and functionality of patients.

## 3. Perspectives and Possibilities of Using Nanomedicine in the Context of Cardiovascular Diseases

Nanomedicine is defined as a branch of science dealing with the application of nanotechnology for the treatment, diagnostics, monitoring, and control of biological systems. It is based on the creation of nanoparticles—structures of 1–100 nm in size with a diverse chemical structure and physicochemical properties. The possibility of the precise control of NC formation processes allows for the adjustment of parameters such as shape, surface charge, or stability in the biological environment, enabling the prediction of NC behavior in vivo. Additionally, a high surface-to-volume ratio determines high load capacity, and the possibility of surface modification allows for the precise delivery of NC to selected structures and molecular targets. Improving the pharmacokinetics of drugs transported by NC allows for the effective use of substances with unfavorable properties—such as low solubility in water, poor bioavailability, rapid metabolism, or serious side effects resulting from systemic and toxic effects [11]. The dynamic development of nanomedicine and subsequent therapeutic successes resulting from its use have contributed to the expansion of the use of nanotechnology to other fields of medicine. Due to their small size and the possibility of labeling, nanoparticles have also been successfully used for imaging as highly sensitive and precise molecular probes. Currently, nanotechnology is still most widely used in the field of oncology, but it has the potential to be used in the diagnosis and treatment of cardiovascular diseases, constituting a promising strategy for the development of targeted therapies and non-invasive methods of imaging pathological changes [12]. Research on liposomal doxorubicin included studies on novel formulations, treatment combinations, and its use in various cancer types. One study explored a new liposomal formulation (TLD-1) in advanced solid tumors. Another research paper focused on combining liposomal doxorubicin with other therapies, such as in breast cancer neoadjuvant treatment. Furthermore, studies have investigated its efficacy and safety in different cancer types, including primary mediastinal large B-cell lymphoma, peripheral T-cell lymphoma, and solid tumors in pediatric patients [13].

## 4. Nanotechnology in the Diagnosis of Cardiovascular Diseases

### 4.1. Detection of Biomarkers of Cardiovascular Diseases

Early CAD diagnostics, especially in acute conditions (e.g., acute coronary syndromes in the form of myocardial ischemia), plays a key role in terms of effective treatment, improves prognosis, and prolongs survival. Currently, laboratory diagnostics are based on the detection of biomarkers of myocardial cell damage, which are released into the bloodstream during ischemia. The most frequently determined markers using mass spectrometry include cardiac troponins (cTns), myoglobin (Myo), creatinine kinase MB (CK-MB), and C-reactive protein. However, these classic detection methods are characterized by limited sensitivity and specificity, especially in determining very low concentrations of these markers in plasma, which can significantly delay diagnosis [14]. For this reason, there is a clear need to develop simple and fast, but at the same time very sensitive, methods of blood sample analysis for markers of myocardial damage, which would enable the faster detection of abnormalities at a very early stage. In this way, it would be possible to implement a treatment that protects against further damage and necrosis of cardiomyocytes.

Nanotechnology offers exciting possibilities for biosensor development but also presents several limitations. Key challenges include scalability, reproducibility, safety concerns, high costs, and ethical considerations. These factors impact the widespread adoption of nanobiosensors, particularly in real-world applications like disease diagnosis and environmental monitoring. The high cost of some nanomaterials, like graphene derivatives and noble metals, further hinders scalability. Achieving consistent performance and reliable results across different batches of nanobiosensors is crucial for their practical use, but this can be difficult to ensure. Some nanomaterials used in biosensors can be unstable in biological environments, affecting their sensitivity and detection limits (Table 1).

Nanotechnology offers numerous advantages across various fields due to its ability to manipulate materials at the atomic and molecular level. These advantages include enhanced material properties, improved energy production and storage, advancements in medicine, and more efficient manufacturing processes. Nanomaterials can be significantly stronger and more durable than their bulk counterparts. For example, carbon nanotubes and graphene can be used to create lighter, stronger, and more flexible materials. Nanomaterials have a larger surface area-to-volume ratio, which makes them more effective catalysts for chemical reactions. This can be beneficial in various applications, including fuel production and chemical synthesis. Nanotechnology can be used to create more efficient electrical conductors, which can be used in electronics and energy storage. Nanomaterials can be designed with specific pore sizes, making them effective filters for water purification and other separation processes. Various nanoparticles, such as nanotubes, nanowires, or nanorods, provide a number of useful properties. The high payload of nanoparticles with a very sensitive reaction ensures ultra-low detection limits [15]. Biosensors usually consist of two parts—a biological element responsible for recognizing the target and a transducer that allows for the molecular event to be converted into a signal that can be recorded—most often electrochemical, optical, or piezoelectric.

Protein targets require prior recognition by specific detection molecules, which may be antibodies, aptamers, or polymers whose task is to selectively bind to antigens. The detection of bound molecules occurs using quantitative methods, including electrochemistry, electrochemiluminescence, fluorescence, and colorimetry, which can be much easier and more accurate due to the optical properties of nanomaterials. For example, the research team of Liyanage et al. designed and described the effectiveness of the first label-free biosensor based on a gold, triangular nanoprism (AuTNP) for the determination of cardiac troponin T (cTnT) in human biological fluids (plasma, serum, urine). The results of the studies showed that this method allows for an attomolar (10^−18^ M) detection limit to be achieved, which makes it at least 50 times more sensitive than standard methods [16]. Such high sensitivity results, among others, from the high surface-to-volume ratio of AuTNP and the porous structure of the nanomaterial, which enable the transport and presentation of numerous recognition molecules (antibodies, aptamers) and transducer elements, which results in cascade amplification of the signal [17].

**Figure 2:**
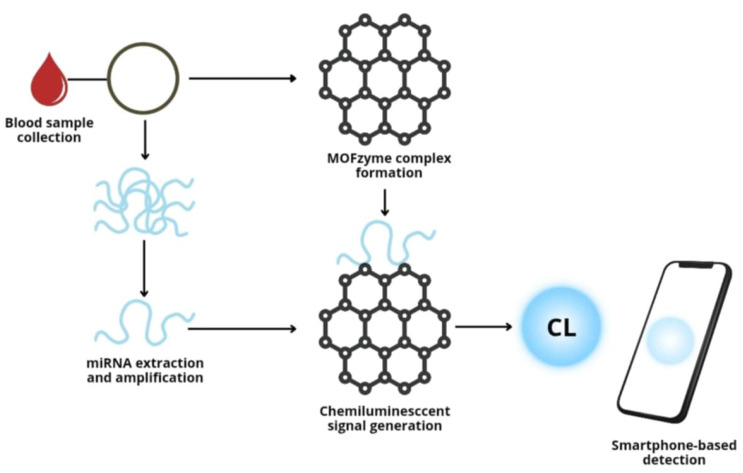
Schematic of double-amplified miRNA detection using spin amplification, MOF with hemin, and G-quadruplex (G4) DNAzymes. The chemiluminescence signal generated by G4/MOFzyme in the luminol–H_2_O_2_ reaction is recorded using a smartphone.

In recent years, research has been underway on the use of other, alternative biomarkers to protein elements. New targets may be non-coding RNA molecules, especially microRNAs (such as miR-144a and miR-499), which play an important role in the pathogenesis of acute coronary syndromes. In the work of Lan MI et al., a new detection platform based on metal–organic frameworks (MOFs) integrated with hemin was presented, enabling the ultrasensitive detection of microRNA (miRNA) using chemiluminescence (CL) in blood samples (Figure 2). In the MOF structure, hemin plays the role of a ligand, giving it catalytic activity similar to peroxidase. The MOF surface allows for the binding of a large number of polymeric G-quadruplex DNAzymes (G4), generated in the amplification reaction dependent on the presence of the target miRNA. The resulting G4-MOF complexes (so-called MOFzymes) act as supercatalysts in the luminol/H_2_O_2_ reaction, generating an intense CL signal.

This platform enables the detection of miRNAs associated with acute myocardial infarction (AMI) in human serum with a sensitivity of up to 1 fM and the ability to read the signal using a smartphone. In addition to a significant improvement in sensitivity, the advantages of this method include great simplicity, no need for advanced equipment, and ~50 times longer CL emission time compared to previous smartphone-based systems [18]. Nanomaterial-based biosensors, therefore, offer fast, precise, and relatively simple methods for determining biomarkers in both blood and other body fluids, but further research is needed to introduce this technique into everyday clinical practice.

### 4.2. Imaging Modality for Nanotechnology Used in CVD

**Figure 3:**
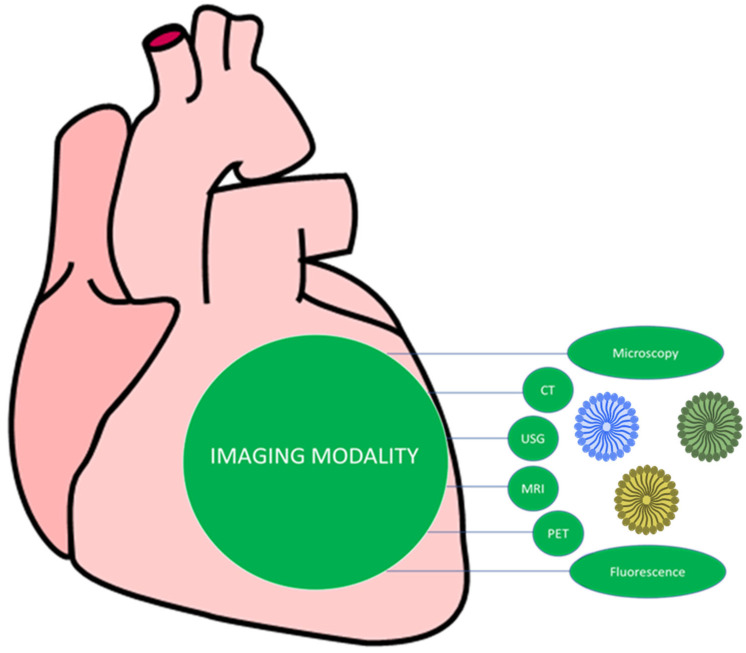
Emerging nano-based techniques with potential to enhance CAD treatment and diagnosis.

Nanotechnology revolutionizes CVD diagnostics by enhancing traditional imaging modalities like MRI, CT, and PET with nanoparticle-based contrast agents for greater sensitivity in detecting atherosclerotic plaques and vulnerable cardiac biomarkers. Emerging nano-based techniques, such as optical coherence tomography enhanced with nanoparticles and nanobubbles for targeted imaging, enable multimodal imaging and real-time tracking during therapy, creating a theranostic approach for simultaneous diagnosis and treatment. Enhanced Traditional Imaging Techniques (Figure 3). Nanoparticles can act as contrast agents, improving the visibility of specific tissues and plaques in magnetic resonance imaging (MRI) and computed tomography (CT) scans. This enables the detection of proinflammatory macrophages and other components of vulnerable atherosclerotic plaques [18]. Radioactive nanocarriers can be used with positron emission tomography (PET) or single-photon emission computed tomography (SPECT) to visualize inflammation and disease progression in coronary arteries [19]. Nanotechnology allows for the creation of multimodal imaging platforms that integrate different imaging techniques into a single vehicle, providing a more comprehensive view of the cardiovascular system. Nanomaterials enhance the sensitivity of biosensors and imaging techniques to detect early signs of CVD and target vulnerable plaques. Nanoparticles can be engineered to target specific biomarkers, damaged tissues, or plaques, improving the accuracy and effectiveness of diagnostic procedures. The development of multifunctional nanoparticles enables theranostics, where imaging and therapy are combined into a single nano-vehicle, allowing for simultaneous diagnosis, real-time tracking during therapy, and targeted treatment.

#### 4.2.1. Nanoparticles in Magnetic Resonance Imaging

Magnetic resonance imaging is one of the key non-invasive imaging techniques used in many fields of medicine. Its great advantage is the ability to visualize abnormalities in the walls of blood vessels with atherosclerotic plaques and thrombi. There are two magnetic resonance angiography techniques: TOF (time of flight) angiography—in 2D or 3D—and phase contrast angiography—PC (phase contrast). Using high-resolution MR devices, it is possible not only to visualize the atherosclerotic plaque itself but also to determine the volume of the lipid core, the presence of hemorrhagic foci, and the thickness of the fibrous cover [19]. Additionally, it is possible to assess the active inflammatory process taking place within the plaque, which allows for the risk of its rupture to be determined. Despite its many advantages, MRI is characterized by lower sensitivity compared to nuclear techniques, hence the need to design sensitive and selective probes capable of accumulating in diseased areas, which would allow for the increased precision of this imaging examination.

Nanoparticles, due to their ultra-small size and the possibility of functionalizing their surface with specifically targeted structures, are currently an attractive research direction. Applying specific biomolecules to organic or inorganic NCs allows for methods to be significantly efficiency for localizing atherosclerotic plaques. However, this is not the only way to increase the accumulation of contrast agent in the lumen of damaged vessels.

**Figure 4:**
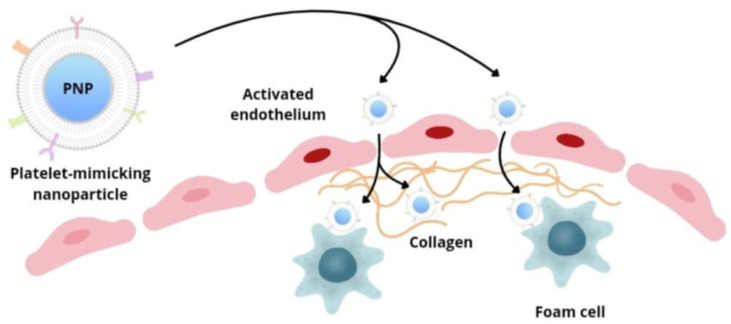
Biomimetic nanocarriers mimicking platelet function localize to sites of atherosclerosis by interacting with activated endothelium, collagen, and foam cells.

Platelets are involved in various stages of atherogenesis—they react to the inflammatory process in the vascular endothelium and participate in the recruitment of inflammatory cells. Zhang et al.’s group developed platelet membrane-coated nanoparticles (PNPs), containing lipid-chelated gadolinium (Gd), capable of targeting many biological elements important for the development of atherosclerotic plaque (Figure 4). Biomimetic nanoparticles are able to interact with activated endothelial cells, foam cells, and collagen and can therefore localize within well-developed atherosclerotic plaques, as well as in areas of arteries at increased risk of their development. This strategy, in addition to providing contrast, also enables information to be obtained on the biology of the target environment, which allows for the detection of both advanced and early atherosclerotic changes at a subclinical stage, as well as the assessment of the degree of progression or regression of changes.

#### 4.2.2. Nanoparticles in Radionuclide-Based Molecular Imaging

Radionuclide-based molecular imaging is widely used to detect atherosclerotic lesions due to its high sensitivity, functional detection capability, and non-invasive nature. Positron emission tomography (PET) allows for 2–3 times higher spatial resolution compared to single-photon emission computed tomography (SPECT) and enables the detection of picomolar concentrations of nuclides.

Initially, PET with 18F-fluorodeoxyglucose (18F-FDG) was proposed as a non-invasive “gold standard” in identifying atherosclerotic plaques, which allows for the detection of inflammation in the vessel walls and identification of areas of macrophage accumulation [20]. However, limitations of this method, such as low spatial resolution and non-specific uptake of the tracer by the myocardium, prompted researchers to search for more specific and selective radiotherapeutics capable of precisely imaging pathological vascular changes.

Radionuclide imaging, also known as nuclear medicine imaging, is a medical imaging technique that uses small amounts of radioactive substances (radionuclides) to diagnose and monitor various diseases. It works by detecting the radiation emitted by the tracer as it accumulates in specific tissues or organs. This allows doctors to visualize the function and structure of these areas, aiding in diagnosis, staging, and treatment planning. Radionuclide safety focuses on minimizing radiation exposure from radioactive materials, adhering to the principles of time, distance, and shielding. These principles are crucial for protecting individuals working with or near radionuclides. The key aspects of radionuclide safety are time (limit the time spent near a radiation source), distance (maximize the distance from the radiation source, as radiation intensity decreases with distance), and shielding (use appropriate shielding materials (e.g., lead, concrete) to absorb radiation). Cancer is the major effect of concern from radionuclides. Radium, via oral exposure, is known to cause bone, head, and nasal passage tumors in humans, and radon, via inhalation exposure, causes lung cancer in humans. Uranium may cause lung cancer and tumors of the lymphatic and hematopoietic tissues.

In order to overcome these limitations, the use of radiolabeled nanoparticles targeting molecular targets has been proposed. A potential biological target of NC may be chemokine receptors, some of which are specifically regulated in macrophages and play a key role in the development of atherosclerotic plaque. An example is the viral macrophage inflammatory protein-II (vMIP-II), targeting chemokine receptors, which was conjugated to a biocompatible, amphiphilic nanoparticle. After radiolabeling with the copper isotope 64Cu, biofunctional nanoparticles sensitively and specifically detected chemokine receptors in both the blood vessel damage model and the mouse atherosclerosis model [21]. Another potential molecular target for imaging atherosclerotic changes is natriuretic peptides and their receptors. Studies have shown that the C-type natriuretic receptor (clearance receptor) may be a biomarker of early pathological changes. Therefore, polymeric nanoparticles were conjugated with different amounts of CANF natriuretic peptide (0%, 5%, 10%, 25%). After radiolabeling and PET imaging, all variants of (64)Cu-CANF-comb NCs showed a more favorable biodistribution profile, including reduced accumulation in the liver and spleen, compared to non-targeted (64)Cu-comb particles. The highest targeting sensitivity and specificity were achieved using 25% (64)Cu-CANF-comb, indicating the potential of this formation as a PET contrast agent for the detection and monitoring of atherosclerosis progression [22].

#### 4.2.3. Nanoparticles in Optical Imaging

Optical nanoparticles are most often used in the optical molecular imaging of neoplastic lesions, but in recent years, their use in the diagnosis of cardiovascular diseases has also been growing. This is due to the high spatial–temporal resolution and high sensitivity of optical methods compared to other imaging techniques. Optical techniques allow for the differentiation of pathological changes at the subcellular level, which allows for the assessment of different stages of atherosclerotic plaque progression. This is possible by directing optical nanoparticles to different molecular epitopes, which allows for the distinction of plaques susceptible to rupture from those that are stable [23]. However, the limited depth of light penetration (from fractions of a millimeter to several centimeters) and the undesirable overlap of the autofluorescence phenomenon in atherosclerotic plaque tissues limit the potential use of optical imaging platforms in the clinic. To overcome this limitation, a new approach has been developed using fluorescence in the second near-infrared window (NIR-II, 1000–1700 nm), allowing for deep tissue penetration and high image resolution [24].

#### 4.2.4. Nanoparticles in Multimodal Imaging

Each of the individual imaging methods has its advantages but also numerous limitations (Table 2), which is why the idea of multimodality has become an important trend in the development of imaging technology. Combining multiple methods allows for obtaining a complementary diagnostic image, and in many cases, this combination significantly exceeds the benefits of a single method. Such a strategy allows for the much more precise detection of cardiovascular diseases [25].

The first multimodal method was the combination of PET and MRI using multimodal nanoparticles. The combination of the high resolution provided by magnetic resonance with deep tissue penetration and the high sensitivity of PET made this method a valuable addition to the toolbox for detecting abnormalities in blood vessels [26]. The design of advanced molecular imaging methods can offer many versatile possibilities for visualizing specific biological targets such as inflammatory infiltration, atherosclerotic plaque formation, thrombosis, or fibrosis.

Dynamic development has made it possible to detect changes at a very early stage, which, in the future, may translate into faster treatment and the more effective prevention of complications related to cardiovascular diseases. However, introducing these advanced molecular imaging methods into everyday practice remains a big challenge. It requires a number of further in vivo studies to be performed that would allow for a precise assessment of the biocompatibility, pharmacokinetics, and, above all, safety of the nanomaterials used. Nanoparticles (NPs) offer benefits like advanced drug delivery, diagnostics, enhanced product performance (e.g., sunscreens), and environmental remediation, but they pose risks such as cellular damage, oxidative stress, inflammation, and organ deposition due to their small size and high surface area, which facilitate crossing biological barriers and interacting with cellular components. The exact toxicity depends on their unique physical and chemical properties, necessitating further research to fully understand their mechanisms of harm and develop effective mitigation strategies.

## 5. Nanoparticles in the Modern Treatment of Circulatory System Diseases

Modern treatment for cardiovascular disease (CVD) involves a combination of lifestyle changes, medications (like statins, antiplatelets, and blood pressure regulators), innovative surgical and minimally invasive procedures (such as stents, angioplasty, bypass surgery, and valve replacements), and advanced technologies like wearable devices for monitoring and artificial intelligence (AI) for personalized care. So far, in everyday clinical practice, patients with circulatory system diseases have not been treated using nanoparticles. However, due to the great hopes associated with this technology, as well as the successes achieved with its use in oncology, it is expected that the process of incorporating NC into the treatment of cardiovascular diseases will be significantly accelerated. Further studies demonstrate the significant benefits of using nanomaterials, which, thanks to their properties, are able to significantly increase the chemical stability and improve the pharmacokinetic profile of transported pharmaceuticals, which makes them a particularly useful tool in therapy aimed at treating CADs.

Nanoparticles are revolutionizing circulatory system treatment by acting as targeted delivery systems for drugs and imaging agents, enhancing the efficacy of therapies for conditions like atherosclerosis, thrombosis, and heart failure, and enabling advanced diagnostics through theranostic approaches. Their small size allows for improved solubility, controlled release, and site-specific delivery of various therapeutic agents, leading to better treatment outcomes with fewer side effects.

Due to the greater ability of NC to accumulate in diseased areas, it is possible to use smaller doses of medicinal substances, which in turn translates into a reduction in their systemic toxic effects and a reduction in—or even elimination of—adverse effects [27]. Nanomedical technologies enable the design of NCs with targeting ligands, enabling the simultaneous detection of atherosclerotic plaques susceptible to the rupture and delivery of a therapeutic agent in a single procedure as an example of the theragnostic approach—giving nanotechnology enormous potential in the modern treatment of cardiovascular diseases.

### 5.1. Nanotechnology in Inhibiting Inflammation in the Atherosclerotic Plaque Microenvironment

**Figure 5:**
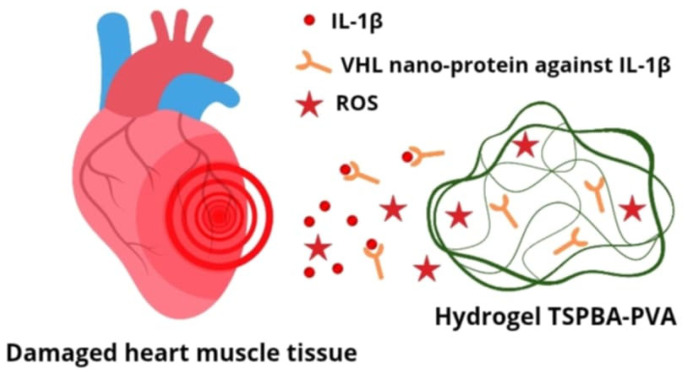
TSPBA-PVA hydrogel loaded with VHH nanoparticle targeting IL-1β and scavenging reactive oxygen species (ROS) at the site of cardiac tissue injury.

Atherosclerosis is a chronic inflammatory process, during which the inflammatory cascade is activated and a number of cytokines are released, which are potential targets for cardiovascular disease therapy. One of the key cytokines of significant importance in this process is interleukin 1 beta (IL-1β), which participates in both acute and chronic inflammatory responses. It has been proven that the level of IL-1β protein and mRNA in patients with atherosclerosis is significantly higher compared to healthy individuals. Additionally, a positive correlation has been demonstrated between the level of this cytokine and the stage of disease progression. It has also been shown that the variability in genes encoding IL-1β may be responsible for the inherited tendency to develop coronary artery disease [28]. Currently, in clinical practice, systemic neutralization of IL-1β itself is used with monoclonal scavenger antibodies, such as canakinumab (blocks the interaction of IL-1β with its receptor, inhibiting the production of inflammatory response mediators). An alternative and promising approach is to target the therapy to earlier stages of the synthesis of this cytokine. An example of such an approach is a study in the field of rheumatology, in which a hybrid lipid–polymer nanoparticle loaded with siRNA against IL-1β was used. This design enabled effective delivery of the therapeutic to macrophages, inhibition of the inflammatory process, and alleviation of arthritis symptoms in a mouse model [29]. Another innovative solution, developed in the context of myocardial regeneration after an ischemic episode, is the use of a nanoprotein (VHH) targeting IL-1β, enclosed in a TSPBA-PVA hydrogel (Figure 5). This hydrogel not only acts as a carrier but also demonstrates the ability to neutralize reactive oxygen species (ROS), which allows for the simultaneous use of two therapeutic strategies in the fight against the effects of oxidative stress.

ROS play a dual role in CVD; at normal levels, they are essential for signaling, but excessive levels, known as oxidative stress, damage cellular components and promote the development and progression of heart and vascular diseases. ROS contribute to atherosclerosis by oxidizing LDL, lead to ischemia–reperfusion injury through mitochondrial damage, and cause cardiac remodeling and arrhythmias by affecting calcium handling and inducing apoptosis in heart cells. ROS uptake protects cardiomyocytes from apoptosis and promotes macrophage transformation towards a regenerative M2 phenotype. Additionally, selectively reacting hydrogels facilitate precise drug release at the site of damage, allowing for achieving local therapeutic concentrations while minimizing side effects. In turn, the use of IL-1β-VHH nanoprotein inhibits the inflammatory cascade at the site of damaged heart tissue. Studies conducted in an animal model have shown that this complex therapeutic strategy not only inhibits inflammation but also supports cardiomyocyte regeneration, which confirms its potential in the treatment of myocardial infarction [30].

### 5.2. Nanocarriers Transport

Nanoparticles transport conventional drugs by encapsulating them to improve solubility, stability, and targeted delivery to specific cells or tissues, thereby reducing side effects and increasing therapeutic efficacy. These nanocarriers overcome biological barriers, facilitate controlled drug release over time, and offer advantages like sustained action and reduced dosage needs. Various types of nanoparticles, including polymeric nanoparticles, lipid nanoparticles, and magnetic nanoparticles, are used depending on the drug and target, demonstrating their growing importance in revolutionizing drug delivery systems for diseases.

Statins are small-molecule drugs that are inhibitors of 3-hydroxy-3-methylglutaryl-coenzyme A (HMG-CoA) reductase, commonly used to reduce the risk of cardiovascular events. Although their efficacy in primary and secondary prevention has been well documented, chronic systemic use may lead to adverse effects such as hepatotoxicity or rhabdomyolysis. Intolerance to statins, leading to the discontinuation of therapy or non-adherence to recommendations, significantly reduces their clinical effectiveness [31]. In response to these limitations, various types of nanocarriers have been developed that enable targeted drug delivery, which allows for the increased efficacy of therapy while reducing its toxicity. An example of such an approach is nanocarriers composed of a bioactive polymer—hyaluronan (HA)—containing atorvastatin in the core (HA-ATV-NPs). They are characterized by the ability to selectively accumulate within atherosclerotic plaques, which results from the property of HA to bind to the CD44 receptor, which is overexpressed on the surface of macrophages within atherosclerotic lesions. In vitro studies have shown that HA-ATV-NPs have a stronger anti-inflammatory effect on macrophages compared to atorvastatin alone, suggesting that targeting the CD44 receptor with HA-ATV-NPs is a promising strategy that allows for the use of traditional lipid-lowering drugs while significantly reducing the side effects resulting from their systemic action [32].

Cholesterol is the main component of atherosclerotic plaques and, together with macrophages, is largely responsible for the inflammation occurring within them. The effectiveness of statins and other antiatherosclerotic drugs is limited due to their low affinity for cholesterol molecules. To overcome this problem, charge-switchable nanoparticles (CSNPs) were designed, the structure of which allows for the selective binding of cholesterol and release of the drug precisely in the microenvironment of the atherosclerotic plaque. CSNPs contain cyclodextrin and simvastatin, enclosed in a phospholipid shell. Cyclodextrin, due to its ability to form complexes with hydrophobic molecules, has a high affinity for cholesterol, but its systemic use in high doses in animals caused serious adverse effects, such as hearing loss due to damage to hair cells in the hearing apparatus. Studies in a mouse model of atherosclerosis have shown that systemically administered CSNPs effectively target atherosclerotic plaques, allowing for the preferential accumulation of the drug within the lesion while eliminating the side effects caused by free-form cyclodextrin. This allows for a synergistic effect resulting from the therapeutic action of simvastatin and targeting with cyclodextrin [33]. A significant obstacle to the clinical use of nanoparticles transporting statins, as well as other drugs, is their elimination as a result of uptake and removal by the reticuloendothelial system (RES) before reaching the site of action. To prevent NC destruction by RES, nanoparticles capable of reacting with reactive oxygen species (ROS) were developed and covered with a macrophage membrane (MM-NPs), which transported atorvastatin in their core (MM-AT-NPs). This biomimetic system not only allows for avoiding elimination by RES but also allows for the precise, ROS-dependent release of atorvastatin at the site of inflammation. Additionally, the macrophage membrane sequesters proinflammatory cytokines, which helps to suppress the local inflammatory response. The synergistic anti-inflammatory and therapeutic effects of such a constructed system result in the increased efficacy of atherosclerosis treatment [34]. Other drugs, such as rapamycin and paclitaxel, which inhibit macrophage migration, smooth muscle cell proliferation, and neovascularization, are also used in atherosclerosis therapy. Rapamycin, an mTOR inhibitor, has unique anti-atherosclerotic effects—it reduces the number of macrophages, induces autophagy, and supports cholesterol efflux from atherosclerotic plaques. However, its common side effect is dyslipidemia, resulting from lipid storage disorders and increased lipolysis, leading to increased LDL levels [35]. In response to the problems with toxicity and low bioavailability of rapamycin, the synthesis of a therapeutic complex based on paramagnetic iron oxide nanoparticles (NPs), low pH-sensitive cyclodextrin and rapamycin, conjugated with profilin-1 (PFN1) antibody, has been proposed. Profilin-1 is present in large amounts on the surface of smooth muscle cells (VSMC), which accumulate within atherosclerotic plaques; therefore, the use of anti-PFN1 antibodies allows for the precise targeting of NCs to diseased areas. Additionally, the presence of paramagnetic iron oxides allows for the imaging of these changes using magnetic resonance imaging (MRI), combining diagnostic and therapeutic functions. Such a designed nanoparticle meets the assumptions of theragnosis, which can significantly increase the effectiveness of atherosclerosis treatment [36].

### 5.3. The Interaction Between Nanoparticles and Immune System

Nanoparticles escape the immune response primarily by employing surface coatings like polyethylene glycol (PEG) to become “stealthy” or by mimicking host cell components, thus delaying opsonization (marking for destruction) and phagocytosis by immune cells like macrophages. Other strategies include using natural cell membranes to cloak nanoparticles, hitchhiking on circulating immune cells to avoid detection, or engineering nanoparticles that actively suppress immune responses rather than trigger them. The goal is to extend their circulation time in the bloodstream and reach their intended targets, such as tumors, without being prematurely cleared by the immune system. Despite their high biocompatibility, nanoparticles are recognized by immune system cells as foreign structures, which leads to their capture and destruction. Therefore, it has become necessary to develop strategies to avoid the reaction of the host’s immune system. One of the solutions being studied is the use of biomimetic coatings, i.e., membranes that replicate natural cell membranes—for example, macrophage or platelet membranes, as mentioned earlier. This approach allows for the “masking” of NCs and increases their ability to circulate in the blood for a long time [37]. In the developing atherosclerotic plaque, there is an intensive expression of adhesion molecules and proinflammatory cytokines, which results in the local accumulation of leukocytes. Therefore, biomimetic nanoparticles based on leukocyte membranes, so-called leukosomes, loaded with rapamycin, were developed. Nanocarriers constructed in this way demonstrate the ability to precisely target the areas affected by inflammation, and at the same time, thanks to the leukocyte membrane, they are less recognized as foreign structures. This allows for the effective delivery of the therapeutic substance without premature elimination by the immune system. Studies have shown that leukosomes with rapamycin effectively inhibit macrophage proliferation and reduce the level of proinflammatory cytokines, which leads to the regression of atherosclerotic lesions [38].

### 5.4. Nanotechnology in Vascular Treatment

Nanotechnology significantly advances vascular treatment by enabling highly targeted diagnostic imaging, improving drug delivery to specific lesion sites, and creating new therapeutic strategies for conditions like atherosclerosis, vascular anomalies, and restenosis. Nanoparticles can carry drugs to reduce plaque, deliver contrast agents for enhanced imaging (MRI, CT), and facilitate tissue repair via nanostructured scaffolds, ultimately improving treatment efficacy and minimizing side effects.

**Figure 6:**
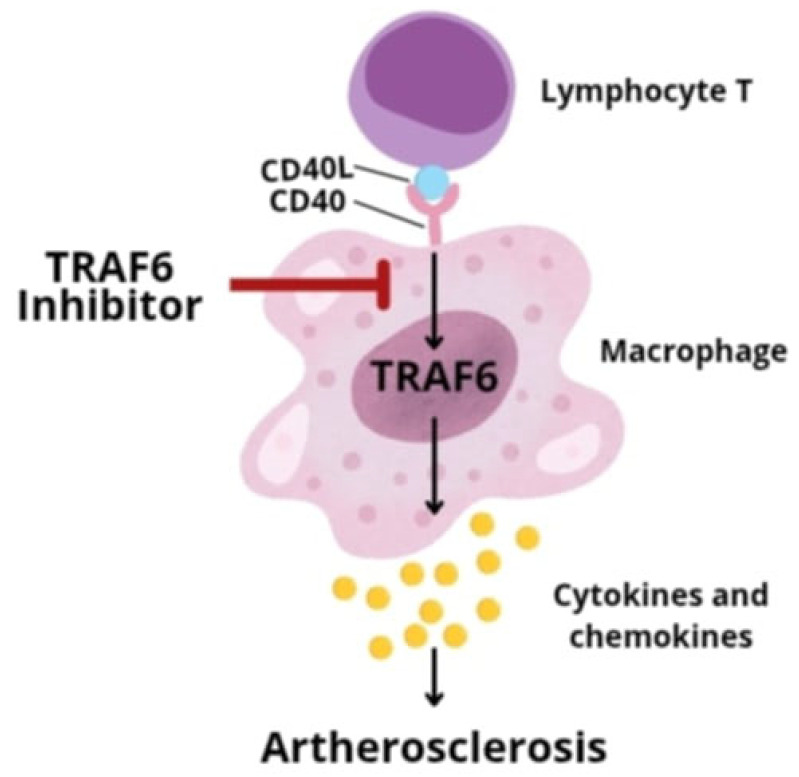
Nanoimmunotherapy targeting CD40-TRAF pathway to reduce atherosclerosis.

Understanding the molecular processes leading to the development of atherosclerosis has allowed for the development of new chemical substances (inhibitors/agonists) targeting individual stages of this process. It has long been known that monocytes and macrophages play a key role in the progression of atherosclerotic changes. They are responsible for the recruitment of inflammatory cells, the accumulation of macrophages, and the secretion of proinflammatory cytokines, which drives the chronic inflammation in the blood vessel wall. Additionally, it has been proven that the accumulation of macrophages itself is directly related to the destabilization of the plaque and the increased risk of its rupture. Therefore, a promising therapeutic strategy may be the use of inhibitors that weaken the activity of monocytes and macrophages. One of the key mechanisms regulating the process of their recruitment and differentiation is T lymphocyte signaling through the interaction of CD40 ligand with CD40L and the activation of TRAF family proteins, in particular TRAF6 (tumor necrosis factor type 6). Initially, attempts were made to use inhibitors blocking the CD40 receptor itself. However, it has been shown that long-term inhibition of this molecule—although effective in terms of antiatherosclerotic activity—leads to undesirable immunosuppression and an increased risk of thromboembolism. This is due to the disruption of signaling by other TRAF family proteins, such as TRAF2, TRAF3, and TRAF5, which are important for producing a proper immune response. Therefore, selective inhibitors blocking the interaction between the CD40 molecule and the TRAF6 factor (TRAF-STOP) have been developed, which do not interfere with the processes conditioned by CD40-TRAF2/3/5, and therefore do not affect CD40-conditioned immunity (Figure 6) [39]. Recombinant high-density lipoprotein (rHDL) nanoparticles were used for the targeted delivery of TRAF-STOP.

Studies have shown that TRAF6i-HDL nanoparticles are selectively taken up by monocytes and macrophages, but they are not internalized by lymphocytes. This led to a decrease in CD40 expression and inhibition of inflammatory cell recruitment, which resulted in the regression of atherosclerotic lesions. Additionally, TRAF-STOP treatment did not disrupt the “classical” immune pathways conditioned by CD40, which confirmed the safety of this therapy [40]. The rapid reduction in inflammation within the atherosclerotic plaque and the favorable toxicity profile emphasize the potential of this method in the treatment of atherosclerosis [41]. Autophagy is a physiological process in which unnecessary or damaged cytoplasmic components, such as organelles or protein aggregates, are spontaneously degraded. This process allows for maintaining the balance of energy in the cell microenvironment, especially when exposed to stressful conditions such as nutrient deficiency, hypoxia, or oxidative stress [42]. Recent reports have shown that macrophage autophagy is associated with many important processes such as lipid metabolism and proinflammatory cytokine secretion, which directly affects the stability of atherosclerotic plaque; hence, the interest in the mechanism of this process has increased in the context of identifying a new therapeutic target for the treatment of atherosclerosis. SIRT1 is a NAD+-dependent histone deacylase protein belonging to the sirtuin family that exerts significant protective effects by inducing autophagy [43]. It has been proven that the administration of SIRT1 inhibitors in a mouse model of atherosclerosis causes the accelerated progression of pathological changes as a result of the increased infiltration of macrophages into the atherosclerotic plaque and disruption of autophagy processes [44]. SIRT1 is also an important regulator in the case of vascular smooth muscle cells (VSMC)—it limits their migration from the media to the intima and also prevents their modification to an inflammatory phenotype [45]. In order to enable the precise delivery of the SIRT-1 activator to the environment of atherosclerotic plaques exposed to disintegration, nanoparticles based on human albumin (HSA) were designed, containing the following:

Fluorescent payload—indocyanine green (ICG) dye;

SIRT1 activator—SRT1720;

Modified peptide molecules targeting osteopontin (OPN).

Diallyl trisulfide (DATS) is a compound capable of generating hydrogen sulfide (H2S) in a controlled and gradual manner through reaction with reduced endogenous glutathione (GSH) [46]. A challenge with its use has been the poor solubility of DATS, which requires a carrier for transport. A controlled H2S release system from DATS was developed, based on mesoporous silica nanoparticles (MSNs) with glutathione (GSH), which allowed for stable drug loading and release in response to specific triggers. In an animal model, DATS-MSN was shown to persist in plasma for at least 9 hours and did not cause significant changes in blood pressure or heart rate, confirming its safety. Furthermore, DATS-MSN is characterized by high biocompatibility, potent cytoprotective activity, and effective protection of the myocardium from damage resulting from ischemia and reperfusion. These results indicate the great potential of this strategy in further research on modern systems of controlled H2S delivery as a therapy for cardiovascular diseases [47].

### 5.5. Nucleic Acid Transporting Nanoparticles

#### 5.5.1. siRNA-Based Therapies

Due to the overexpression of osteopontin in activated VSMCs with an inflammatory phenotype, nanoparticles specifically accumulated within atherosclerotic plaques after their intravenous introduction in an animal model. The effective delivery of the SIRT1 activator had a beneficial effect on the morphology of sensitive plaques, which was mainly due to the inhibition of the phenotypic change in smooth muscle cells—one of the key factors of atherosclerotic progression. After two weeks of therapy, an improvement in the size and composition of the plaques was noted, which suggests the increased efficacy of SRT1720 delivered in the nanoparticle form compared to the free form. These results indicate that theragnostic nanoparticles designed in this way have great potential for the simultaneous identification and targeted treatment of unstable atherosclerotic lesions [48]. Currently, intensive studies are also underway on the potential use of SIRT1 in protecting the myocardium during pathology, especially ischemia and subsequent reperfusion. The activation of autophagy during the ischemic phase allows for the removal of excess metabolic waste and promotes cardiomyocyte survival, but excessive autophagy during reperfusion can lead to the depletion of damaged cell reserves, then their autophagic death, and, consequently, to an increase in the area of myocardial damage [49]. Therefore, SIRT1-mediated autophagy plays different roles in the individual stages of myocardial damage and regeneration; hence, a better understanding of this process in the future may allow for the development of new drugs capable of its precise modulation. There is growing interest in the therapeutic potential of controlled and gradually released gas molecules, such as H2S and NO. Nitric oxide (NO) is a well-known vasodilator, but its clinical use is limited by its short half-life and lack of targeting damaged tissues [50]. To overcome these limitations, nanocarriers capable of targeted NO transport have been designed. HDL lipoprotein mimicking particles were constructed, consisting of a 5 nm gold core (AuNP) with a surface functionalized with apoprotein A-I and covered with a phospholipid bilayer, which allowed for the self-assembly of synthetic S-nitrosylated phospholipid (SNO). The obtained carrier—SNO-HDL-NP—combines the functionality of HDL and NO, demonstrating beneficial effects in the treatment of atherosclerosis and reducing ischemic and reperfusion injury in a mouse kidney transplant model. These results confirm the effectiveness of transporting a therapeutic dose of NO and the potential of HDL-patterned nanocarriers also for other therapeutic molecules [51]. In parallel, studies are being performed on the therapeutic use of hydrogen sulfide (H_2_S), which shows a protective effect in the course of prolonged ischemia, especially in the myocardium [52]. However, the clinical use of H_2_S is limited by its toxicity at high concentrations and insufficient control over its release. The most commonly used donor—sodium hydrosulfide hydrate—is characterized by low stability and rapid, uncontrolled gas release. For this reason, intensive work is underway to develop systems enabling the stable, controlled, and targeted delivery of H_2_S to diseased tissues [53].

**Figure 7:**
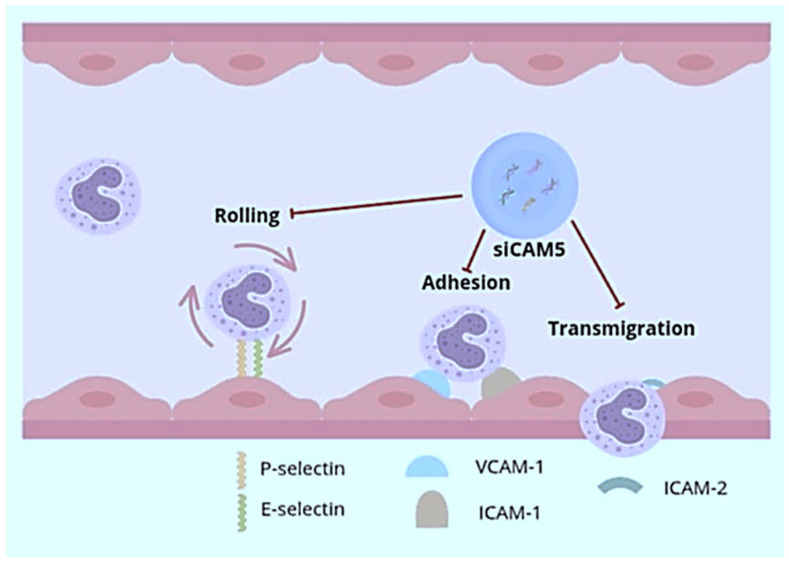
siCAM5 inhibiting subsequent steps in leukocyte migration, rolling, adhesion, and transmigration, by reducing the expression of adhesion molecules.

Gene therapy is currently a rapidly developing therapeutic strategy that shows great potential in the treatment of cardiovascular diseases. One of the promising methods is the use of small interfering RNAs (siRNAs) for therapy, which are double-stranded RNA molecules capable of selectively silencing the expression of genes with a homologous sequence, thus enabling the inhibition of the production of a specific protein [54]. siRNAs are of great interest due to their high efficacy, low therapeutic doses, and lack of the need for frequent administration of the therapeutic agent. Despite the great potential of this method, its application is limited due to the difficulties regarding the safe and targeted delivery of these molecules, especially to extrahepatic tissues. Currently, all approved siRNA-based drugs target the liver, which is due to the high blood flow and natural ability to filter and capture drugs of this organ [55,56]. A number of strategies based on nanocarriers have been developed for the safe and effective delivery of siRNAs. One of the potential therapeutic targets for siRNA in atherosclerosis is the c-Jun N-terminal kinase (JNK) pathway, which belongs to the MAPK kinase family. In particular, JNK2—one of the three isoforms—plays a key role in the proapoptotic response to cellular stress and in the progression of atherosclerotic changes. The activation of JNK2 by scavenger receptors promotes macrophage recruitment, lipoprotein uptake, and foam cell formation. Due to the lack of selective JNK2 inhibitors, an attempt was made to develop an siRNA nanocarrier specifically for this isoform that is capable of targeted action within atherosclerotic plaques. The developed system is based on a flexible carrier composed of a cationic peptide, which demonstrates the ability to detect local pH changes and enables effective escape of therapeutic molecules from the endosome [57]. It has been shown that nanostructures designed in this way rapidly penetrate damaged endothelial barriers within the atherosclerotic plaque, leading to a local decrease in JNK2 expression, which results in the attenuation of prothrombotic activity, the restoration of endothelial barrier integrity, and a decrease in the number of macrophages in the plaque while there is no immunological reaction or toxicity to other tissues. Nanostructures designed in this way can be used not only to deliver JNK2-specific siRNAs but also as a universal platform for other therapeutic RNA molecules, which makes them a promising tool in gene therapy in many fields of medicine [58]. Loading nucleic acids into nanoparticles enables the simultaneous transport of different siRNAs, which is a new, attractive therapeutic approach, allowing for precise limitation of the translation process of several protein targets simultaneously. Nanocarriers transporting siRNAs, referred to as siCAM5, have been developed, which allow for a reduction in the expression of five endothelial cell adhesion molecules (CAMs). CAMs, together with chemokines and their receptors, are responsible for the recruitment of neutrophils and monocytes to sites of ongoing inflammation. The process of leukocyte recruitment is multi-step, including rolling (mediated by E- and P-selectins), retention and adhesion (involving vascular cell adhesion molecule 1—VCAM-1 and intercellular adhesion molecules—ICAM-1), and migration mediated by ICAM-2 [59]. In order to inhibit this process, siRNAs targeting ICAM-1 and ICAM-2, VCAM-1, and E- and P-selectins were created, which were encapsulated in polymeric nanoparticles (Figure 7).

The applied treatment reduced leukocyte recruitment and inflammation within the atherosclerotic plaque in a mouse model of atherosclerosis and after myocardial infarction. Additionally, the reduced infiltration of immune cells into acutely ischemic myocardium improved cardiac tissue regeneration and reduced the area of damage. These studies suggest the possibility of the effective, parallel targeting of multiple genes as a therapeutic route to inhibit the progression of atherosclerosis and prevent complications after ischemic myocardial injury [60]. The activation of specific signaling pathways within macrophages may contribute to many disease processes, in particular to the development of atherosclerosis [61]. Ca^2+^/calmodulin-dependent protein kinase γ (CaMKII γ) has been found to play a major role in the development of thin, necrotic atherosclerotic lesions. Macrophage CaMKIIγ has been shown to be activated in advanced human and murine atherosclerotic plaques, where it promotes the conversion of relatively benign, fibrous atherosclerotic lesions into necrotic lesions with thin fibrous caps at high risk of rupture. Studies suggest that this mechanism is due to CaMKIIγ suppression of the ATF6/liver X-receptor α (LXRα)/c-Mer proto-oncogene tyrosine kinase (MerTK) pathway, thereby impairing efferocytosis. Disruption of this process results in the secondary necrosis of damaged cells and ultimately leads to increased inflammation through necrotic changes within the atherosclerotic plaque, which weakens the overlying fibrous cap and increases the risk of plaque rupture [62]. Nanoparticles containing CaMKII γ-siRNA inhibiting the expression of the gene encoding CaMKII γ, composed of PLGA polymer, were designed, which through S2P—a peptide recognizing the macrophage receptor stabilin-2—were targeted to these cells [63]. Nanocarriers tested in a mouse model of atherosclerosis caused a significant reduction in CaMKII γ expression, increased MerTK activity, improved the phagocytosis of apoptotic cells (efferocytosis), reduced the area of the necrotic core, and increased the thickness of the fibrous cap, which altogether increased the stability of the atherosclerotic plaque. The results of these studies suggest the possibility of future use of therapies reducing the risk of complications resulting from acute thrombosis of the vessel lumen due to atherosclerotic plaque rupture by stabilizing atherosclerotic plaques sensitive to disintegration [64].

#### 5.5.2. miRNA-Based Therapies

Among the RNA molecules, microRNAs (miRNAs) also find their place in medicine. miRNAs are non-coding, single-stranded RNA molecules capable of regulating the expression of other genes; for example, by inhibiting their translation or degrading target mRNAs [65]. Many of the endogenously occurring miRNAs can serve as markers of pathological processes; for example, in the case of myocardial ischemia, an increase in circulating molecules such as miR-18a, miR-27a, miR-30e, miR-26b, miR-199a, miR-106a, and miR-652 was observed [66]. Additionally, designing synthetic miRNAs can help to inhibit many pathological processes, including the progression of cardiovascular diseases. However, similarly to siRNA therapy, therapeutic activities based on miRNA are also limited due to the tendency to degrade these molecules, the induction of an immune response, and a lack of targeted action. Nanoparticle-based drug delivery systems, due to their efficiency and precise targeting capabilities, may solve this problem. Chitosan nanoparticles (chNPs) capable of delivering functional miRNA mimics (miR-33) to macrophages have been developed to regulate the expression of the target gene ABCA1. ATP-binding membrane cassette transporter A1 (ABCA1) is crucial for cellular cholesterol efflux to apoA1 and for reverse cholesterol transport (RCT), a process responsible for the transport of excess cholesterol from peripheral tissues to the liver via high-density lipoproteins [67]. Studies have shown that in the case of the use of miRNA inhibiting ABCA1 expression in a mouse model, cholesterol uptake to apoprotein A1 and reverse cholesterol transport (RCT) to plasma, liver, and feces are reduced, while in the case of miRNA promoting ABCA1 formation, the expression of this protein and cholesterol efflux to RCT were significantly improved. The results of these studies demonstrate that miRNAs can be effectively delivered to macrophages via chitosan nanoparticles (chNPs) to regulate ABCA1 gene expression [68,69]. In order to better target nanoparticles, carriers capable of recognizing individual receptors were designed; for example, the CC2 chemokine receptor (CCR2), which is expressed on vascular smooth muscle cells (VSMCs) with an inflammatory phenotype capable of migration and proliferation. It has been shown that the VSMC phenotype switch is regulated by microRNA-145 (miR-145). Therefore, micelles with miR-145 targeting the CCR2 receptor were synthesized [70]. In a mouse model of early-stage atherosclerosis, one dose of miR-145 micelles prevented lesion growth by 49% and maintained an increased level of miR-145 expression 2 weeks after treatment. Additionally, miR-145 micelles inhibited atherosclerotic plaque growth by 35% and 43%. It has been demonstrated that the CCR2-targeted nanoparticle-mediated delivery of miR-145 to VSCM can attenuate the development of atherosclerosis by inhibiting cell proliferation within the atherosclerotic plaque [71].

## 6. Nanocarriers with Therapeutic Properties

Designed nanoparticles are used for the targeted and thus more effective delivery of various therapeutic agents to the atherosclerotic plaque environment. However, in recent years, attention has been paid to the possibility of creating nanocarriers that could exhibit therapeutic activity themselves. Considering the key role played by reactive oxygen species (ROS) in the pathogenesis of atherosclerosis, nanomaterials with intrinsic antioxidant and anti-inflammatory activity may be a promising therapeutic approach in the treatment of cardiovascular diseases [72]. Nanoparticles capable of eliminating ROS were developed and synthesized by the covalent coupling of a mimetic agent—superoxide dismutase and a hydrogen peroxide-eliminating compound—phenylboronic acid pinacol ester with a cyclic polysaccharide—β-cyclodextrin (TPCD). In these studies, TPCD nanoparticles constructed in this way were rapidly and selectively internalized by macrophages and vascular smooth muscle cells (VSMC), which led to the elimination of intracellular ROS and attenuation of oxidative stress-induced inflammation and limitation of cell apoptosis. The use of TPCD allowed for stabilization of atherosclerotic plaques—a smaller number of cholesterol crystals, a reduction in the volume of the necrotic core, thickening of the fibrous cap, and a decrease in the number of macrophages and the activity of matrix metalloproteinase MMP-9 were observed, which reduced the risk of complications associated with atherosclerotic plaque rupture. The therapeutic benefits resulted mainly from a reduction in systemic and local oxidative stress, and thus inflammation, which determined a smaller infiltration of inflammatory cells into the plaque environment. In addition, preliminary in vivo tests have shown that TPCDs are well tolerated during long-term intravenous administration, making them a potential therapeutic agent for wide application in the treatment of atherosclerosis and other cardiovascular diseases [2]. In addition to polymeric nanomaterials, inorganic nanocarriers, especially those based on metals, have properties that slow down the progression of atherosclerosis. An example is selenium, which reduces oxidative stress, modulates the inflammatory response, protects endothelial cells from dysfunction, and prevents apoptosis and calcification of vascular cells [73]. However, the intake of selenium and other metals is limited due to the narrow therapeutic window significantly reducing the safety of the therapy. To solve this problem, selenium-enriched Astragalus polysaccharide nanoparticles (Se-APSs) have been developed, which show high biological activity, bioavailability, and, at the same time, low toxicity and controlled release. The results of these studies suggest the possibility of the future use of metal nanoparticles in the prevention and treatment of cardiovascular diseases, as well as in other chronic diseases such as arthritis, cancer, hypothyroidism, or viral diseases [74].

**Figure 8:**
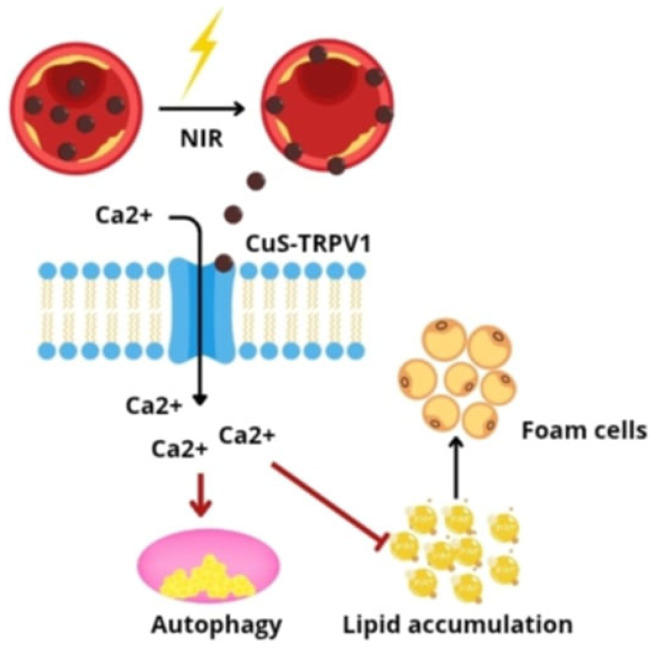
Activation of CuS-TRPV1 nanoparticles by NIR induces Ca^2+^ influx, promoting autophagy and inhibiting lipid accumulation to reduce foam cell formation.

In recent years, there has been increased interest in the possibility of using nanotechnology in photodynamic therapy (PDT). The principle of PDT is based on three components: photosensitizer, light, and molecular oxygen. The exposure of photosensitive compounds to a specific type of light catalyzes the formation of singlet oxygen, which exerts a cytotoxic effect on diseased cells. Currently, the use of PDT is mainly focused on superficial changes, especially cancerous ones, which results from limitations related to the delivery of photosensitizers to diseased sites (low solubility, lack of selectivity) and the depth of light penetration into tissues. In response to these limitations, new photosensitizer nanocarriers have been developed, which increase the effectiveness of the PDT method through better accumulation in target tissues [75]. A classic photosensitizer—Chlorin E6 (Ce6), with poor tissue penetration ability—was conjugated with silica nanoparticles (UCNPs), creating the UCNP-Ce6 compound. This system is characterized by good hydrophilicity, biocompatibility, and favorable optical properties [76]. The experimental results have shown that PDT via UCNP-Ce6 promotes cholesterol efflux by activating the autophagy process, induced in part by generating reactive oxygen species (ROS) under the influence of photosensitization and activating the ROS/PI3K/Akt/mTOR pathway, which proves that PDT can be used as a therapeutic strategy in the treatment of atherosclerosis [77]. It has been proven that the activation of TRPV1 cation channels by capsaicin can reduce lipid storage and slow down the formation of atherosclerotic lesions, but the use of this compound is limited due to toxicity developing during its chronic use. Adverse effects result, among others, from metabolic processes occurring in the liver, as a result of which metabolites are formed that are capable of accumulating and inducing neoplastic processes [78]. An alternative method of stimulating TRPV1 channels is to use antibodies specific for these channels, which are additionally conjugated with copper sulfide (CuS) nanoparticles. The system developed in this way, under the influence of near-infrared radiation (NIR), acts as a photothermal activator of TRPV1 in vascular smooth muscle cells (VSMCs). Exposure to NIR causes a local increase in temperature, which leads to the opening of thermosensitive TRPV1 channels and the influx of Ca^2+^ ions into the cells. The increased concentration of intracellular Ca^2+^ induces autophagy and inhibits the formation of foam cells in response to the action of oxidized low-density lipoproteins (oxLDL) (Figure 8).

In an in vivo model, the use of the CuS-TRPV1 conjugate enabled the high-resolution, photoacoustic imaging of coronary vessels and significantly reduced lipid accumulation and atherosclerotic plaque development in mice. These results confirm the potential of this strategy as a precise and controlled therapy modulating TRPV1 signaling pathways in the treatment of atherosclerosis [79].

## 7. Conclusions

Nanotechnology, due to the enormous diversity of designed structures and functions, shows exceptional potential in the diagnosis and treatment of cardiovascular diseases. The use of nanoparticles enables the development of highly sensitive and precise biosensors, significantly reducing the diagnostic time. Compared to conventional imaging methods, targeted nanocarriers can selectively accumulate within atherosclerotic plaques, increasing the sensitivity and accuracy of detecting pathological changes.

In CVD therapy, nanotechnology enables the implementation of various treatment strategies, including the repair of damaged endothelium, anti-inflammatory, antioxidant effects, or the inhibition of platelet aggregation. Most often, NCs act as multifunctional drug carriers, increasing their bioavailability, protecting against degradation, and enabling targeted delivery of therapeutics to the microenvironment of atherosclerotic plaque. In recent years, attention has also been paid to the natural therapeutic properties of some nanomaterials, such as the ability to neutralize ROS and photothermal or photodynamic effects, which additionally expand their application.

Despite numerous benefits, the widespread implementation of nanotechnology in clinical practice still faces significant challenges. Further, well-designed preclinical and clinical studies are necessary to provide reliable data on the pharmacokinetics, toxicity, and long-term safety of nanocarriers. It is also necessary to understand the interactions of NCs with other therapeutic compounds and elements of the biological environment.

A key challenge also remains the development of industrial methods for the production of nanocarriers with controlled and repeatable physicochemical properties while maintaining economic profitability. Fulfilling these conditions is a necessary condition for introducing nanotechnology as a standard method in modern cardiovascular medicine.

## Figures and Tables

**Table 1:** The biosensor advantages and disadvantages.

Advantages	Disadvantages
Increased solubility of highly lipophilic drugs	Lack of proper knowledge about the effect of nanoparticles on biochemical pathway and processes in human body
Tunable physical and chemical properties	Unpredictable genotoxicity due to insufficient toxicological assessment studies
Targeted drug delivery	Carcinogenesis
Drug release in a sustained and controllable manner	Elimination and metabolism vary with different types of materials used in nanoparticles synthesis
Good biocompatibility and bioavailability	More expensive

**Table 2:** Imaging method comparison.

Imaging Method	Advantages	Disadvantages
MRI (**magnetic resonance imaging**)	high spatial resolution	low sensitivity
NIFR (**nuclear–infrared fluorescence**)	high sensitivity low background signal low cost	insufficient tissue penetration depth
PET (**positron emission tomography**)	very high sensitivity	low spatial resolution exposure to ionizing radiation

## Data Availability

Not applicable.
